# Muscle and Bone Defects in Metastatic Disease

**DOI:** 10.1007/s11914-022-00741-y

**Published:** 2022-08-22

**Authors:** Martina Pauk, Hiroaki Saito, Eric Hesse, Hanna Taipaleenmäki

**Affiliations:** 1 Institute of Musculoskeletal Medicine, University Hospital, LMU Munich, Munich, Germany; 2 Musculoskeletal University Center Munich, University Hospital, LMU Munich, Munich, Germany

**Keywords:** Metastasis, Muscle weakness, Cachexia, Bone destruction, Secreted factors

## Abstract

**Purpose of Review:**

The present review addresses most recently identified mechanisms implicated in metastasis-induced bone resorption and muscle-wasting syndrome, known as cachexia.

**Recent Findings:**

Metastatic disease in bone and soft tissues is often associated with skeletal muscle defects. Recent studies have identified a number of secreted molecules and extracellular vesicles that contribute to cancer cell growth and metastasis leading to bone destruction and muscle atrophy. In addition, alterations in muscle microenvironment including dysfunctions in hepatic and mitochondrial metabolism have been implicated in cancer-induced regeneration defect and muscle loss. Moreover, we review novel in vitro and animal models including promising new drug candidates for bone metastases and cancer cachexia.

**Summary:**

Preservation of bone health could be highly beneficial for maintaining muscle mass and function. Therefore, a better understanding of molecular pathways implicated in bone and muscle crosstalk in metastatic disease may provide new insights and identify new strategies to improve current anticancer therapeutics.

## Introduction

Metastasis is a multi-step process, which includes dissemination of cancer cells from the primary tumor, survival in the circulation and colonization at distant metastatic site. Bone metastases are most common in patients with prostate and breast cancer, and to a lesser extent in kidney, lung, and thyroid cancer patients [1,2]. Bone metastatic cascade is initiated by cancer cell invasion through the basement membrane and extracellular matrix and migration to the blood or lymphatic system [3]. During cancer cell invasion, important roles are attributed to matrix metalloproteinases (MMPs) due to their ability to cleave and degrade extracellular matrix (ECM) [4]. In addition, cancer cells undergo epithelial-mesenchymal transition (EMT) which enhances their invasiveness, while following homing at metastatic site they revert to epithelial phenotype by mesenchymal-epithelial transition (MET) required for metastatic outgrowth [5]. Once in the bone, metastatic cancer cells secrete cytokines including parathyroid hormone-related peptide (PTHrP), which induce bone-forming osteoblasts to produce excessive amount of receptor activator of nuclear factor kappa-B ligand (RANKL) to activate the bone-resorbing osteoclasts. Upon bone resorption, growth factors such as transforming growth factor β (TGF-ß) that further stimulate tumor growth creating a so-called “vicious cycle of bone metastasis.” In addition to osteolytic lesions, certain cancers, such as prostate cancer, result in osteoblastic metastases due to abnormal formation of woven, poor-quality bone.

Although skeletal muscle is the most abundant tissue in the vertebrate body, metastases are very rare with a prevalence range from 0.03 to 17.5% [6]. However, excessive muscle wasting, or the loss of muscle tissue, is commonly observed in metastatic cancer [7,8]. The release of soluble proteins, exosomes, and metabolites from metastatic tissues can systematically affect distant organs such as muscle and lead to muscle wasting syndrome, also known as cachexia [7,9]. Cachexia is a multi-organ wasting syndrome characterized by ongoing loss of skeletal muscle mass, decreased muscle strength, systemic inflammation, and increased basal energy expenditure that cannot be fully reversed by conventional nutritional support [10,11]. Ultimately, cachexia leads to progressive functional impairment, decreased quality of life, and increased mortality of cancer patients [12]. In general, skeletal muscle wasting has been highly associated with pancreatic, stomach, colorectum, lung, head-neck, and breast cancer patients [13]. Currently, there is no standard treatment for cancer cachexia. Thus, there is an urgent need for further investigation and better understanding of underlying mechanisms which could lead to potentially new therapeutic targets.

## Muscle Defects in Metastatic Disease

### Molecular Mechanisms Underlying Cancer Cachexia

The mechanisms that drive metastasis-induced cachexia are not fully understood. While muscle protein breakdown in cancer is clearly induced, changes in muscle protein synthesis are not consistent [14,15]. The main protein degradation pathway is the ubiquitin-proteasome system, which involves muscle specific E3 ligases atrogin-1/MAFbx (muscle atrophy F-box protein) and MuRF-1 (muscle-specific RING-Finger-1) [14]. In advanced cancer, transcriptionally activated E3 ligases mediate ubiquitination of muscle structural and contractile proteins, thus contributing to muscle atrophy and decreased muscle function [14]. Muscle degradation in cancer is also mediated through the autophagic-lysosomal system, which induces lysosomal-dependent degradation of cytoplasmic proteins and organelles, and calcium-dependent proteolysis composed of cysteine proteases, also known as calpains [14–16]. In metastatic cancer, these pathways are activated by pro-inflammatory cytokines such as interleukin 6 (IL-6), interleukin 1 (IL-1), tumor necrosis factor alpha (TNF-α), and interferon gamma (IFN-γ) [17–20]. These cytokines, secreted by cancer cells, immune cells, and other non-cancer cells within the tumor microenvironment, contribute to systemic inflammation and activate catabolic processes in muscle through transcriptional regulators such as p38 MAPK, nuclear factor kappa B (NF-κB), and STAT3 [11,17]. In addition to cytokines, other tumor-derived factors are implicated in cancer cachexia such as hormones, metal ions, microRNAs, and members of the TGF-β superfamily including TGF-ß, activin A, growth differentiation factor-11 (GDF-11), and myostatin [17,21,22]. Also, recent studies suggest that alterations in the muscle microenvironment in cancer cachexia can affect its regenerative ability [23,24]. Furthermore, mitochondrial dysfunction in skeletal muscle has been implicated in muscle catabolism and cancer-induced muscle wasting [25].

### Mechanisms Underlying Muscle Defects in Bone Metastases

Metastatic bone disease is often associated with skeletal muscle weakness [22,26–28]. Osteolytic bone metastases stimulate bone resorption, which leads to the release of bone-derived factors, particularly TGF-β, from the bone matrix [22,29]. The mechanism by which TGF-β contributes to skeletal muscle weakness was shown to be through NADPH oxidase 4 (NOX4)-mediated oxidation of skeletal muscle proteins such as ryanodine receptor/calcium (Ca2+) release channel (RyR1) [22]. As RyR1 channels are required for muscle contraction by releasing calcium from sarcoplasmic reticulum stores into cytoplasm, their oxidation results in intracellular calcium leakage, which leads to muscle weakness. A similar mechanism was observed in mouse models of osteolytic bone metastases from breast, prostate, and lung cancers; multiple myeloma; and a syngeneic mouse model of osteolytic cancer in the bone [22,28]. Consistently, we have demonstrated the effect of osteolytic breast cancer bone metastases on muscle weakness and muscle fiber atrophy via bone-matrix–derived TGF-ß and activated p38/NF-κB signaling cascade [27••]. Pharmacological inhibition of sclerostin an inhibitor of the WNT signaling and bone formation reduced cancer progression and bone destruction and importantly improved muscle microarchitecture and function. In addition, observed expansion of Pax7-positive satellite cells in the muscle most likely contributes to declining muscle strength, as NF-κB activation usually leads to quiescent state of satellite cells and dysregulation of the myogenic program [30]. The regenerative capacity of muscle was partially restored by anti-sclerostin antibody treatment, which resulted in suppressed p38/NF-κB signaling and restored number of Pax7-positive satellite cells [27••].

In accordance with our findings, a severe muscle regeneration defect was associated with an elevated number of Pax7-positive satellite cells in a cancer cachexia model using colon-26 (C26) cancer cells [23,24]. Mechanistically, the impaired myogenesis resulted from a suppressed differentiation potential of satellite cells due to reduced neutrophil infiltration and macrophage recruitment in cachectic muscle or dysregulated IL-4-dependent signaling. Regarding the latter, IL-4 treatment counteracted cachexia and restored muscle mass by increasing muscle protein synthesis [24]. Furthermore, muscle regeneration was partially restored by IL-4 as shown by a reduced accumulation of satellite cells and fibro-adipogenic progenitors, which are non-myogenic muscle stem cells that regulate muscle homeostasis and can differentiate to adipocytes or fibroblasts in pathological and chronic conditions [31]. Finally, IL-4 administration potentially increased the immune response against the tumor as large areas of necrosis in tumors were accompanied by an increased number of cytotoxic lymphocytes and type II macrophages.

### Mechanism of Muscle Defects in Soft Tissue Metastases

Recent studies have identified the role of the metal-ion transporter ZIP14 in both advanced cancer patients and metastatic breast cancer and colon cancer mouse models [7]. ZIP14 was upregulated in cachectic muscles by TGF-ß and TNF-α, leading to enhanced ZIP14-mediated zinc uptake by muscle, reduced expression of myogenic regulatory factors, and loss of myosin heavy chain. Similar mechanism of ZIP14 upregulation and increased zinc uptake in the muscle was associated with the activation of TGF-ß/SMAD signaling and progression of cachexia in metastatic models of breast cancer and pancreatic ductal adenocarcinoma [32,33]. Therefore, ZIP14 provides a potential link between zinc accumulation and metastasis-induced cachexia and serves as a potential therapeutic target.

Cachectic phenotype was also observed in advanced colorectal cancer models, including C26, MC38, and HCT116, accompanied by development of liver metastases [34••, 35, 36]. Muscle atrophy was linked with aberrant activation of STAT3, which contributed to proteolysis pathways through activation of Atrogin-1 and MuRF1 [34••,^35^]. STAT3-mediated muscle wasting was triggered by circulating IL-6, whereas inhibition of IL-6/STAT3 signaling rescued muscle atrophy [35]. Indeed, increased levels in total STAT3 have been shown to correlate with poor prognosis of advanced cancer patients [37]. Recently, HSP90-mediated activation of STAT3 was shown to activate ubiquitin-proteasome pathway in a FOXO1-dependent manner in cancer cachexia [38]. Furthermore, the release of HSP90 by cancer cells was responsible for Toll-like receptor 4 (TLR4) activation and promotion of muscle catabolism by activating p38/MAPK signaling cascade [39], consistent with previous data in pancreatic cancer, where ZIP4-mediated release of HSP70 and HSP90 promoted muscle atrophy [40]. Colorectal cancer progression was accompanied with bone resorption and bone loss; however, the presence of liver metastases exacerbated cachectic phenotype [34••, 35, 36]. Hepatic alterations have been associated with cancer-induced muscle wasting [41,42] and furthermore liver fibrosis-induced muscle atrophy was promoted by elevated levels of circulating IL-6, TNF-α and myostatin during progression of liver disease [43,44].

Recently, altered mitochondrial homeostasis has been implicated in cancer-induced muscle wasting [25,34••,^35^,^45^], contributing to a shift from oxidative to glycolytic metabolism, which results in intramuscular lipid accumulation and decreased muscle strength and function [34••]. Indeed, patients with metastatic melanoma demonstrated fatty infiltration in the muscle, leading to reduced skeletal muscle density and poor survival [46]. Moreover, suppression of PGC1α, the major regulator of mitochondrial biogenesis, has been linked with high circulating IL-6 levels [47] and muscle atrophy [34••,^35^,^45^]. In addition, mitochondrial dysfunction results in release of myokine FGF21 [48], which contributes to muscle loss [49]. Most recently, cachectic muscles have been characterized by reduced mitochondrial iron content accompanied with increased catabolism, while iron supplementation restored mitochondrial function, which resulted in improved muscle mass, function and strength [50]. These results establish a critical role of iron in maintaining skeletal muscle homeostasis, revealing iron metabolism as a potential therapeutic target in cancer-induced muscle atrophy.

Regarding other cytokines, IL-8 which is released from pancreatic cancer cells at high levels correlated with muscle atrophy, acting through CXCR2 receptor and activation of ERK1/2 signaling [51]. More recently, high serum concentration of IL-35 in advanced non-small lung cancer and breast cancer patients was linked with increased skeletal muscle atrophy and activation of MuRF1 and Atrogin-1 [52]. Stress-responsive cytokine GDF15 and its receptor GDNF receptor alpha like (GFRAL) have been implicated in cancer cachexia [53]. Moreover, exosomes secreted by C26 colon cancer cells were enriched with GDF-15 and found to contribute to the development of cancer cachexia by inducing muscle atrophy via regulating Bcl-2/caspase-3 pathways [54].

New cachectic and anti-cachectic factors have been identified in recent years. For instance, PAUF, which is secreted by pancreas cancer cells, functions through Atrogin-1-dependent catabolic pathways and has been associated with poor clinical outcome in pancreatic cancer patients [55]. Furthermore, IFIT2 depletion was shown to induce oral squamous cell carcinoma metastasis and skeletal muscle atrophy through IL-6 signaling [56], whereas deletion of stress-response protein REDD1 prevented chemotherapy-induced muscle atrophy via mTORC1-dependent signaling [57]. 3-MA was recently identified as an anti-cachectic and anti-tumorigenic factor in pancreatic cancer acting through inhibition of p53 apoptosis effector related to PMP22 (PERP) and suppression of pancreatic cancer cell growth [58].

### MicroRNAs in Metastasis-Induced Muscle Weakness

To date, several noncoding RNAs are known to be involved in cancer-mediated muscle wasting [59]. Recently, Xie et al. demonstrated that repression of miR-29c induced leukemia inhibitory factor (LIF) in muscles that promoted muscle wasting through the JAK/STAT and MAP kinase pathways [60]. Muscle atrophy in colorectal cancer patients with metastasis associated with increased circulating levels of miR-203 secreted by metastatic tissue [21]. Mechanistically, overexpression of miR-203 resulted in suppressed proliferation and stimulated apoptosis of skeletal muscle cells via targeting BIRC5 (survivin), a negative regulator of apoptosis. While miR-181a-3p and both miR-195a-5p and miR-125b-1-3p induced muscle atrophy by activating apoptotic signaling pathways [61,62], a decrease in miR-497-5p mediated by IL-6, counteracted muscle atrophy by stimulating expression of hypertrophy-related genes in cancer cachexia [63]. Furthermore, miR-450-5p and miR-451a were found differentially expressed in skeletal muscle of cachectic lung cancer patients; however, more research is needed to better understand their regulation in muscle atrophy [64].

### Models to Investigate Cancer-Induced Muscle Defects

Mechanistic studies of metastasis-induced cachexia have been limited due to the lack of animal models that recapitulate clinical features seen in humans. Therefore, development of new mouse models is needed to improve our knowledge on the mechanisms that drive the disease and possibly provide new therapeutic targets. In line with this, multiple zebrafish models of metastatic hepatocellular carcinoma have been established in recent years [65]. These models exhibit inflammation and cancer-induced skeletal muscle wasting and could thus be useful in high-throughput in vivo screening for anti-metastatic or anti-cachectic drugs. Recently, a new mouse model with metastases to the lungs has been developed by utilizing human papilloma virus (HPV) and oropharyngeal squamous cell carcinoma cell line [66]. This model recapitulates key features of cancer cachexia, as evident by progressive loss of body mass, functional disability, systemic inflammation, and muscle wasting mediated by activation of ubiquitin proteasome and autophagy pathways. A new murine model of breast cancer with spontaneous metastases has been developed recently by orthotopic injection of Bard1-deficient breast cancer cells that spontaneously metastasize to the lung [32]. Affected mice developed cancer-associated muscle atrophy, demonstrating the suitability of this model for future translational research.

### Therapeutic Targeting of Cancer-Induced Muscle Defects

A number of promising drug candidates are being assessed for cancer cachexia [67–72]. Specifically, activin type 2 receptor (ActRIIB) or its ligands such as myostatin, GDF11, and activins are attractive therapeutic targets considering their important role in the regulation of muscle growth. Many myostatin inhibitors failed in clinical trials in recent years; however, new potential candidates such as IMB0901 exhibited promising results in rescuing muscle atrophy in cancer cachexia [73]. Furthermore, inhibition of ActRIIB signaling by ActRIIB-Fc effectively preserved skeletal muscle mass and strength in mice bearing advanced colorectal cancers [74]. Moreover, dual anti-ActRIIA/IIB antibody treatment reduced serum levels of IL-6 and reversed cachexia in mice, supporting a functional link between activin A and IL-6 signaling pathways observed in ovarian cancer cells [75]. In addition, blocking type I receptors ALK4/5 of the TGF-β family preserved cancer-associated muscle loss and downregulated catabolic processes in the muscle [76]. Endogenous antagonist follistatin-like 3 (FSTL3) binds to activins, GDF8, and GDF11 [77], without affecting other ligands of the TGF-β family. Indeed, systemic administration of monovalent human FSTL3 Fc-fusion protein (mono-FSTL3-Fc) resulted in increased muscle mass in mice, representing a promising therapeutic option for muscle loss due to its more specific action and less adverse effects than ActRIIB-Fc [77]. In cancer cachexia, inhibition of Activin A preserved muscle mass and MEF2C expression, which is involved in the regulation of MYHC7 expression, one of the myosin heavy-chain (MYHC) isoforms in muscle [78]. Inhibition of GDF15–GFRAL signaling by monoclonal antibody 3P10 showed beneficial effects on lipid metabolism and reversed cancer cachexia in mice [79]. Anti-cachectic and anti-tumorigenic effects of mitochondrial assembly receptor (MasR) agonist AVE 0991 were identified [80]. In addition, administration of mitochondria-targeting antioxidant mitoquinone preserved skeletal muscle mass and strength, normalized mitochondrial homeostasis, and improved oxidative metabolism, which contributed to decreased intramuscular fat infiltration in cancer cachexia [81•]. Another mitochondria-targeted peptide SS-31 had only partial effect on preventing body wasting, but improved mitochondrial activity and mostly modulated liver metabolome by rescuing the levels of glucose and glycogen that are usually reduced in cachexia[82].

## Bone Defects in Metastatic Disease

### Early Stages of Bone Metastasis and the Pre-Metastatic Niche

Metastasis starts in the primary site and involves cancer cell intrinsic and extrinsic events. Besides changing their phenotype to an invasive, mesenchymal-like through epithelial-mesenchymal transition (EMT), accumulating evidence suggests that primary tumors establish a permissive pre-metastatic niche within the bone by secreting tumor-derived factors such as growth factors and extracellular vesicles (EVs), by modifying extracellular matrix and recruiting bone marrow-derived cells (BMDCs) [83]. Various cytokines such as epidermal growth factor (EGF), hepatocyte growth factor (HGF), platelet-derived growth factor (PDGF), and TGF-ß are involved in EMT induction including transcription factors Snail, Slug, Twist, and ZEB1 [84]. In addition, EMT is associated with suppressed anti-tumor immune response [85]. Recently, oncogene MCT-1 was found to promote IL-6/IL-6R/STAT3 axis that leads to increased EMT process and cancer stemness but also affects the tumor immunity as seen by increased polarization of macrophages toward the immunosuppressive M2 phenotype which drive the invasiveness of breast cancer cells [86]. Tumor-associated macrophages (TAMs), M1 and M2 macrophages, participate in the formation of the tumor microenvironment, immunosuppression, and regulation of tumor growth [87]. Moreover, M2 macrophages were found to secrete chemokine CCL5, which promotes prostate cancer cell invasion, migration, and EMT via activating β-catenin/STAT3 pathway, whereas CCL5 knockdown suppresses tumor growth and bone metastases [88]. Circulating tumor cells escape detection by the immune system and settle within the bone marrow microenvironment, where they interact with the osteoblastic niche through connexin 43 gap-junctions, thereby activating calcium signaling and cancer cell growth in bone [89]. In addition, a key role of CXCL12/CXCR4 signaling axis in mediating cancer cell homing to bone has been previously established [83].

Significant advances have been made over the recent years in understanding the importance of the pre-metastatic niche. Breast cancer cell–derived EVs are recruited by the bone microenvironment where they increase the ability of osteoblasts to secrete cytokines and EVs to induce osteoclast formation and metastasis-induced osteolysis [90]. EVs also transfer miR-21 to osteoclasts and promote osteoclast differentiation via regulating programmed cell death 4 (PDCD4) expression [91•]. Xu et al. showed that novel circRNA circIKBKB promoted breast cancer bone metastases by inducing the bone pre-metastatic niche through NF-κB signaling pathway [92]. Therefore, disrupting the communication between breast cancer cells and the bone microenvironment would present an interesting future therapeutic strategy for bone metastases. Moreover, breast cancer–derived factors support attachment and survival of disseminated tumor cells to the premetastatic niche in bone by inducing changes in bone mineral properties [93]. Similarly, R-spondin 2 (RSPO2) and RANKL, secreted from breast cancer cells, are involved in the recruitment of osteoclast progenitors and formation of osteoclastic pre-metastatic niche [94]. They bind to the LGR4 receptor and regulate the expression of Dickkopf-related protein 1 (DKK1), a soluble inhibitor of Wnt signaling. Recently, cells from the immune system such as dendritic cells were shown to differentiate to osteoclast progenitors in response to T cell–mediated release of cytokines, such as RANKL, while interleukin 23 (IL23) produced by differentiated dendritic cells further maintains T cell pro-osteoclastogenic activity in the bone marrow [95]. This keeps a positive feedback loop of bone destruction and contributes to the formation of the pre-metastatic niche within the bone microenvironment before tumor cell homing.

Disseminated cancer cells can enter an extended period of proliferative dormancy within the bone metastatic niche and become reactivated by escaping cell cycle arrest [96–98]. Dormant breast cancer cells compete with long-term hematopoietic stem cells for the occupancy of the endosteal niche, which is enriched in spindle-shaped N-cadherin+/CD45− osteoblasts (SNOs) and keeps tumor cells in quiescent state in a Notch2-dependent manner [99]. Inhibition of the Notch2 pathway has been suggested to reactivate and mobilize dormant breast cancer cells from the endosteal niche by releasing the interaction between dormant cancer cells and SNOs. Consequently, cancer cells can exit the bone microenvironment and colonize distant sites such as the liver. Recently, N-Cadherin was found to play an essential role in maintaining breast cancer cell dormancy in the bone, by increasing their capacity to adhere to SNOs [100]. Moreover, a group of genes, such as *Cfh*, *Gas6*, *Mme*, and *Ogn*, is highly expressed in dormant breast cancer cells in bone and correlated with recurrence-free survival in breast cancer patients [101]. Disseminated breast cancer cells residing in bone marrow perivascular niche are protected from chemotherapy by their integrin-mediated interactions with molecules including von Willebrand Factor (VWF) and vascular cell adhesion molecule-1 (VCAM-1) [102]. Disruption of these interactions with integrin inhibitors sensitized cancer cells to chemotherapy and reduced bone metastases. Accordingly, integrin β3 has been shown to promote chemoresistance in bone metastases, whereas mTORC1 inhibitor therapy enhanced the chemotherapy effect as evident by decreased bone metastases and cancer-induced bone loss [103].

### Cancer-Induced Bone Destruction

Adult bone is continually remodeled by the processes of bone resorption and bone formation [104]. Following successful seeding of disseminated cancer cells in the bone, tumor cells interact with the bone microenvironment leading to tumor growth, which can elicit osteoclast-mediated bone resorption or osteoblast-mediated bone formation. Most common osteolytic metastases are caused by bone resorption, where cytokines IL-1 and IL-6 and PTHrP and RANKL play crucial role in osteoclast formation and activation [83]. Continuous release of cytokines and growth factors from the bone matrix further supports osteoclast activation and tumor growth via various signaling pathways including the RANK/RANKL/osteoprotegerin (OPG)-axis, canonical WNT, and bone morphogenetic protein (BMP)/TGF-ß signaling pathways [105]. Conversely, osteoblastic metastasis in prostate cancer results from excessive bone formation activated by many factors such as endothelin-1 (ET-1), BMPs, PDGF, and TGF-β [83].

Cancer cells preferentially colonize the trabecular region of bone enriched with osteoblasts and micro-vessels [106]. Cancer cell-osteoblast interactions within the bone microenvironment are essential for bone metastasis progression, as osteoblasts are known to protect breast cancer cells from stress-induced death by both paracrine and juxtracrine signals and therefore can limit the number of cancer-supportive niches [107]. However, Kolb et al. demonstrated that a subpopulation of osteoblasts in the bone microenvironment is involved in the suppression of breast cancer cell growth via decorin and NOV (CCN3) proteins [108]. Furthermore, these osteoblasts produce EVs enriched with miR-148a-3p, which further suppresses bone metastatic breast cancer proliferation partly through extracellular signal-regulated kinase 1/2 (ERK1/2) signaling [109]. We have previously identified an important role of TG-interacting factor-1 (Tgif1) in mediating interactions between breast cancer cells and osteoblasts in the bone marrow microenvironment [110]. Absence of Tgif1 in osteoblasts resulted in suppressed breast cancer cell migration and bone metastases, which is mediated through increased Semaphorin 3E (Sema3E) expression. In addition, acidosis may contribute to the colonization of breast cancer cells in the bone by promoting extracellular matrix (ECM) organization [111] and by interfering with bone remodeling [112]. Acidic environment recruits osteoclast precursors and stimulates osteoblasts to secrete the pro-osteoclastogenic factors RANKL and macrophage colony-stimulating factor (M-CSF) and inflammatory mediators TNF, IL-6, and IL-8, which promote osteolysis. Similarly, ERK1/2 activation in both cancer cells and osteoblasts induced inflammatory phenotypic conversion of osteoblasts leading to secretion of cytokines and growth factors, which promoted osteoclastogenesis and cancer growth. This effect was reverted following ERK1/2 inhibition by trametinib [113].

### Growth Factors Mediating Bone Defects

The bone microenvironment is rich in growth factors, which stimulate tumor growth and metastasis. Recently, it has been demonstrated that tumoral TGF-β signaling has a role in promoting bone metastatic progression and osteolysis in ER+ breast cancer through stimulating the secretion of osteolytic factors such as PTHrP [114]. In addition, TGF-β-induced DACT1 suppressed WNT signaling and promoted breast and prostate cancer bone metastasis [115]. In both prostate and breast cancer, TGF-β was found to stimulate tumor microenvironment by modulating the recruitment of bone marrow-derived mesenchymal stem cells (BMSCs) into the tumor, mediated by transmembrane protein neural cadherin (N-cadherin) [116,117]. More recently, prostate cancer–derived GDF15 was found to increase the osteoclastogenic potential of osteoblasts, which secrete RANKL and CCL2 to promote bone resorption [118]. In addition, prostate cancer cells induced osteocytes to secrete GDF15 into the bone microenvironment which, in turn, stimulated early growth response 1 (EGR1) expression in prostate cancer cells and promoted tumor progression [119]. BMPs are actively involved in the tumor development and bone metastatic progression by mediating interactions between cancer cells and the bone environment [120,121]. Previously, it was reported that conditional deletion of BMPR1a in myeloid cells suppresses prostate tumor growth and changes macrophage polarization [122]. In myeloma bone disease, inhibition of BMP signaling prevented bone loss by reducing osteoclastogenesis and promoted osteoblast differentiation by reducing the concentration of sclerostin the bone marrow [123]. Pharmacologic inhibition of BMP signaling by small molecule antagonist DMH1 in prostate cancer models of bone metastasis restricted cancer cell colonization to bone in immunodeficient mice; however, in mice with intact immune system, DHM1 had no effect on tumor growth and bone health [124]. Interestingly, numerous studies identified a dual role of BMPs in cancer development with BMPs acting both as tumor promoters or suppressors [125]. Recently, inhibition of the BMP pathway by LDN-193189 was shown to enhance bone metastasis development in breast cancer [126], suggesting future studies are needed to elucidate the role of BMP signaling in cancer patient treatment.

## The Role of Inflammation and Immune Suppression in Bone Metastasis

Bone metastasis formation and progression are associated with systemic inflammation and immune suppression [127,128]. Numerous studies have identified key roles of immune cells such as macrophages, neutrophils, dendritic cells, natural killer cells, and T cells in the formation of bone metastatic niche. In advanced breast cancer, monocyte-derived macrophages promote bone metastasis growth in an IL4R signaling-dependent manner [129], indicating that inhibition of macrophages and IL4R may lead to a new potential therapy targeting bone metastasis. Neutrophil infiltration in bone results in enhanced survival of the metastatic cells by weakening cytotoxic CD8+ T cells responses in advanced breast cancer [130]. Mechanistically, downregulation of catenin delta 1 (CTNND1) in metastatic bone lesions promoted tumor recruitment to bone by upregulating CXCL12/CXCR4 axis via PI3K/AKT/HIF-1α pathway, while recruitment of neutrophils in the bone was stimulated by secretion of GM-CSF and IL-8. This was not in accordance with a previous report demonstrating the anti-tumor role of neutrophils in advanced prostate cancer [131]. Costanzo-Garvey et al. showed that neutrophils induce apoptosis of disseminated cancer cells; however, during bone metastasis progression, neutrophils gradually lose the cytotoxic effect on prostate cancer cells. Recently, estrogen-related receptor alpha (ERRα) was shown to inhibit metastasis progression of breast cancer cells by activating immune response in the bone [132]. Recruitment of CD8+ T cells to bone was enhanced by the production of chemokines CCL17 and CCL20, while suppression of TGF-ß, a key repressor of T cell activity, resulted in increased antitumor cytotoxic response in the bone. In addition, eosinophils were found to induce tumor cell migration and metastasis in bone through CCL6-CCR1 signaling [133].

## Secreted Molecules and Extracellular Vesicles in Bone Metastases

Cytokines and chemokines are involved in bone metastatic progression at different stages [134]. IL-11 plays an essential role in breast cancer bone metastases by inducing osteoclastogenesis via JAK1/STAT3 signaling pathway independent of RANKL [135]. Accordingly, blocking of STAT3 activation reduced osteolysis and bone metastatic progression. In addition, neutrophil-derived IL-4 plays a key role in osteolysis in colorectal cancer (CRC) with bone metastases [136]. IL4/IL4Rα signaling activated ERK pathway, which further stimulated the proliferation of osteoclast precursors in bone metastases. Treatment with Ravoxertinib, an inhibitor of the ERK pathway, prevented IL4-mediated bone resorption. Bone marrow–derived IL-1ß stimulates breast cancer metastatic colonization in the bone microenvironment by promoting WNT signaling via NF-κB and CREB [137,138]. Hence, targeting IL-1β-WNT signaling by IL-1 receptor inhibitors such as Anakinra prevented colonization of disseminated cancer cells into bone and decreased the overall metastatic burden. Consistently, formation of the bone metastatic niche was recently demonstrated to be regulated through NAT1/NF-kB/IL-1ß axis [139]. Tulotta et al. demonstrated a dual role of IL1ß, where microenvironment-derived IL-1ß promoted the progression of breast cancer metastases in bone, whereas it inhibited the growth of primary tumor by recruiting innate immune cells with possible anti-tumor roles [140]. Only combined therapy of anakinra with doxorubicin and zoledronic acid exhibited anti-inflammatory effect and markedly inhibited both primary tumor growth and metastatic recurrence in bone. MSC-secreted IL-28 stimulated apoptosis of bone metastatic prostate cancer cells through STAT1 signaling; however, following chronic exposure to IL-28, certain populations of cancer cells became resistant to apoptosis and shifted to STAT3 signaling [141]. Accordingly, STAT3 inhibition resulted in a decreased prostate cancer progression in bone and may be responsible for desensitizing prostate cancer cells to chemotherapy. By using ex vivo bone metastasis culture, chemokine CXCL5 was found to promote breast cancer colonization in bone and could contribute to the switch from a dormant state [142].

Recently, EVs have been identified as important mediators of crosstalk between cancer cells and the bone microenvironment, enabling the transfer of active molecules to distant sites [143,144]. Furthermore, EVs are involved in the formation of the pre-metastatic niche, dissemination of cancer cells to metastatic sites, and cancer cell growth and survival [145]. The role of EVs has been extensively reported in bone metastatic prostate cancer. Prostate cancer–derived EVs target bone marrow cells leading to activation of NF-κB signaling and increased osteoclast differentiation, thereby further enhancing metastatic tumor burden in a cholesterol-dependent manner [146]. Interaction between the long non-coding RNA NORAD and miR-541-3p promoted bone metastases in prostate cancer by upregulating the release of EVs enriched with pyruvate kinase M2 (PKM2) from prostate cancer cells to BMSCs [147]. Moreover, exosomal PKM2 is transferred to BMSCs where it upregulates the production of CXCL12 in a HIF-1α-dependent fashion and subsequently contributes to prostate cancer growth and progression of bone metastasis [148]. In addition, multiple myeloma-derived exosomes are reported to stimulate osteoclastogenesis, acting directly through the IRE1α/XBP1 axis or via amphiregulin (AREG)-mediated activation of EGFR pathway in osteoclast progenitors followed by the release of pro-osteoclastogenic MSC-derived IL-8 [149,150].

Among the biomolecules transported by EVs, non-coding RNAs including lncRNAs and miRNAs exhibit various roles in metastatic bone disease [151,152]. Recent studies demonstrated that several miRNAs are involved in the regulation of osteoclasts and osteoblasts during bone metastasis progression in breast, prostate and colorectal cancer [153–161]. Furthermore, these miRNAs show a correlation with disease progression and can be used as biomarkers for cancer progression [162–166]. In breast cancer, the novel lncRNA DGUOK-AS1 was identified to promote cancer progression and bone metastasis by decreasing tumor suppressor miR-204-5p and stimulating the secretion of IL-11 [167]. In bone metastasis of prostate cancer, novel tumor suppressive miRNAs, miR-582-3p, and miR-582-5p inhibit bone metastases through inactivation of NF-κB signaling [168,169], whereas miR-532-3p and miR-204-5p by suppressing TGF-ß signaling activity [170], suggesting a strong potential as therapeutic target. Moreover, Dai et al. demonstrated the important role of TGF-ß-dependent double-negative feedback loop between miR-33a-5p and ZEB1 in the promotion of prostate cancer bone metastasis [171]. Exosomal miR-378a-3p promoted prostate cancer progression and osteolysis by targeting Dyrk1a/Nfatc1 pathway in bone marrow macrophages leading to increased secretion of angiopoietin like 2 (Angptl2) into the bone microenvironment [172]. Ma et al. demonstrated that prostate cancer-derived EVs deliver miR-152-3p to osteoclasts and promote bone osteolysis by targeting osteoclastogenic regulator MAFB [173]. In hepatocellular carcinoma, lnc34a was identified to promote bone metastasis acting through suppression of miR-34a, which inhibits TGF-ß/Smad signaling and its downstream targets, connective tissue growth factor (CTGF) and IL-11 [174]. In non-small cell lung cancer, exosomal lncRNA-SOX2OT promoted bone metastases by targeting TGF-β/pTHrP/RANKL signaling pathway in osteoclasts [175].

## Novel Approaches to Treat Bone Metastases

Animal models of skeletal metastasis are essential for understanding the pathogenesis of cancer bone metastases. There has been an increased interest in generating new three-dimensional (3D) in vitro models including patient-derived xenograft models, organoid, and scaffold models that can mimic native bone microenvironment [176–185]. In addition, numerous new compounds have been evaluated for their effect and therapeutic potential on metastatic bone disease [186,187,196–198,188–195]. Currently approved therapeutic agents for bone metastases include chemotherapy, radiotherapy, bisphosphonates, and anti-RANKL therapy [199]. Recently, a series of experiments were conducted to apply the technology of induced tumor-suppressing cells (iTSCs) to bone cells, MSCs, and cancer cells, where activation of oncogenic signaling such as WNT results in production of tumor-suppressing secretomes [200]. WNT activation by LRP5/ß-catenin overexpression in osteocytes generated tumor-suppressive secretomes, which successfully suppressed tumor growth and bone destruction by downregulating chemokines CXCL1 and CXCL5, upregulating tumor suppressors such as P53 and suppressing the expression of oncogenic genes such as MMP-9, Runx2, TGFβ, and Snail [201,202]. Similar anti-tumor capability was observed in MSCs generated by overexpressing LRP5, β-catenin, Snail, or Akt [203]. Activation of WNT signaling in osteoclasts, osteoblasts, and cancer cells resulted in their conversion to tumor-suppressive cells with secretomes enriched with Hsp90ab1, enolase 1 (Eno1), moesin (MSN), and ubiquitin C (Ubc), which acted as atypical tumor-suppressors [204–206]. Osteoclast secretome-derived Hsp90ab1 and Eno1 inhibited tumor progression by suppressing TGF-ß signaling and interacting with CD44, facilitating tumor cell killing by natural killer (NK) cells [204,207]. They also exhibited bone-protective roles as osteoclast secretome inhibited RANKL-stimulated osteoclast differentiation and stimulated osteoblast differentiation. Collectively, novel iTSC technology and generation of anti-tumor secretomes represent a potential therapeutic approach for bone metastases. In recent years, a nanoparticle-based drug delivery system (DDS) is often used to deliver different therapeutics to bone [208,209]. Huang et al. showed that nanoparticles loaded with cisplatin and zoledronate significantly inhibited tumor growth and bone resorption in breast cancer bone metastasis [210]. Furthermore, gold clusters suppressed breast cancer-induced osteoclastogenesis and osteolysis, demonstrating their potential for treating breast cancer bone metastases [211].

## Conclusions

**Fig. 1:**
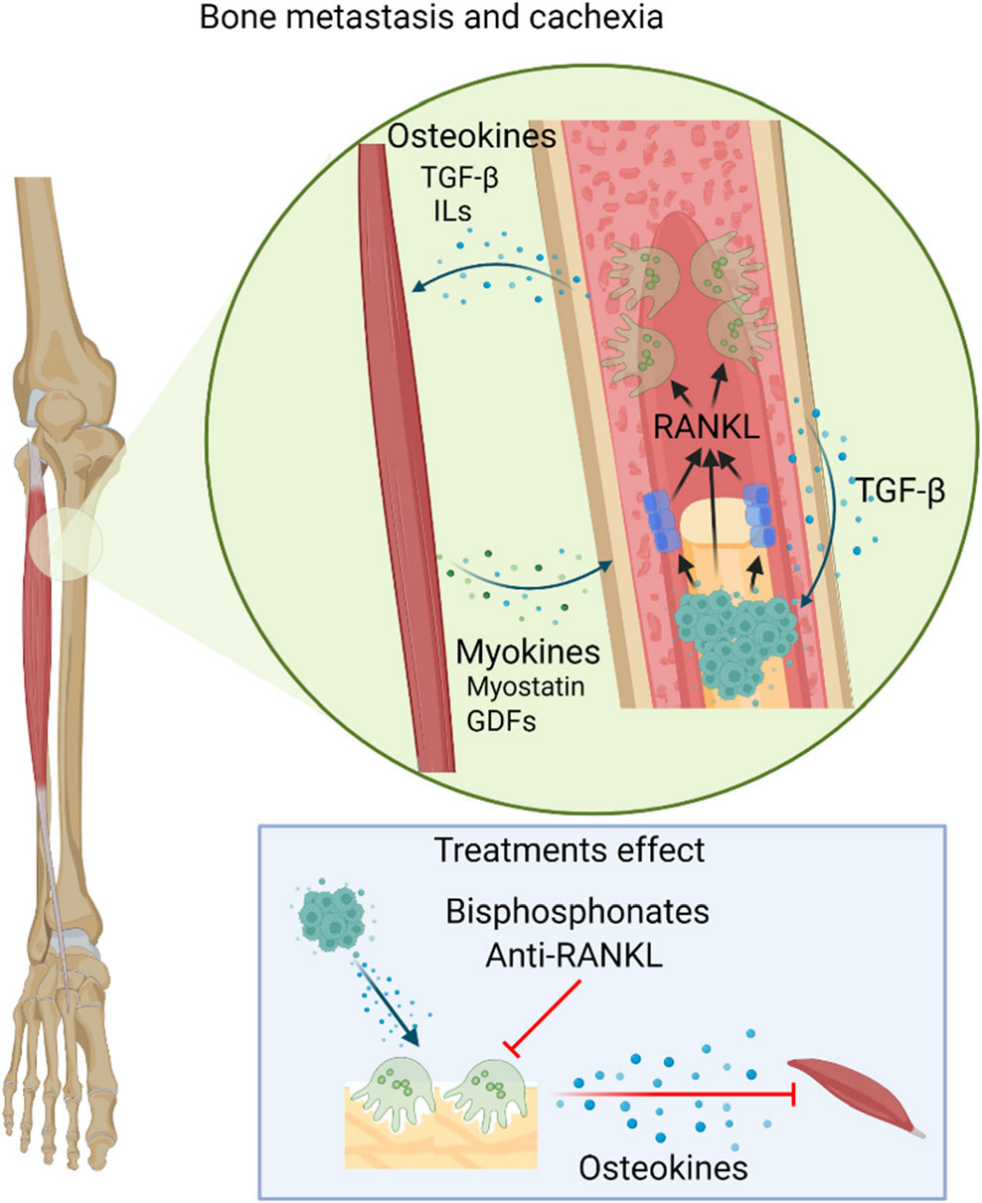
Bone-muscle crosstalk in bone metastasis**.** Bone-derived osteokines and muscle-derived myokines mediate the bone-muscle interactions in physiological and pathological conditions. In bone metastasis, several cytokines are released from the bone matrix (e.g., TGF-β) that impair muscle function and promote tumor growth. Furthermore, local such as RANKL promote bone destruction and reduce muscle strength. Thus, preventing pathological bone resorption could be an effective therapeutic strategy to preserve not only the bone but also muscle health in metastasis

Here, we presented an overview of recent discoveries related to metastatic bone and muscle disease. Muscle and bone share close mechanical and biochemical relationship, giving rise to muscle-bone crosstalk with both tissues releasing either muscle-derived myokines or bone-derived osteokines that positively or negatively affect bone and muscle metabolism (Fig. 1). Hence, pharmacological approaches for bone health could be efficient in preserving muscle mass and function in cancer cachexia. Recently, it was demonstrated that tumor-derived RANKL in cancer-bearing mice is associated with increased bone turnover and skeletal muscle atrophy, while anti-RANKL or bisphosphonate treatment preserved bone and partially prevented the loss of muscle mass and strength [212••]. Moreover, the administration of bisphosphonates in mice exposed to a chemotherapeutic agent had beneficial effect on muscle mass and strength, acting through bone preservation and inhibiting the release of bone-derived factors upon bone resorption [213]. Therefore, a better understanding of molecular pathways implicated in cancer-mediated bone resorption and muscle wasting may provide new insights for discovering new antiresorptive, anti-cachectic and possibly anti-cancer therapeutics.
